# Serological survey on *Sarcoptes scabiei* var. *suis* in fattening pigs and sows from intensive farms in Italy

**DOI:** 10.1007/s00436-026-08692-4

**Published:** 2026-05-09

**Authors:** Luca Villa, Carolina Allievi, Alessia Libera Gazzonis, Susanna Adelaide Sechi, Maria Teresa Manfredi

**Affiliations:** 1 Department of Veterinary Medicine and Animal Sciences, Università degli Studi di Milano, Via dell’Università 6, Lodi, 26900 Italy; 2 Research Laboratory of Animal Parasitic Diseases and Zoonoses (ParVetLab), Università degli Studi di Milano, Via dell’Università 6, Lodi, 26900 Italy

**Keywords:** Sarcoptic mange, Ectoparasite, Swine, ELISA, Biosecurity, Zoonosis

## Abstract

Sarcoptic mange is the clinical disease caused by the burrowing mite *Sarcoptes scabiei*, an obligate ectoparasitic arthropod responsible for significant morbidity in both domestic and wild animals. The aim of the study was to investigate the serological prevalence of *S. scabiei* var. *suis* in pigs raised in the intensive system in Lombardy region, one of the most suitable regions in northern Italy for intensive pig farming, and to assess the influence of variables related to farm management on the exposure to mite infestation. 219 fattening pigs and 151 sows from 23 conventional farms in Lombardy were sampled; data on farm management were collected, and a biosecurity score was determined for each farm. Blood samples were analysed using a commercial indirect ELISA Kit; generalized linear models were developed to determine the influence of production category and sanitary score on the parasite infestation. At the farm level, 65.2% (15/23) of the selected farms were positive, i.e. 90.9% of those housing sows and 40% fattening pigs. At the individual level, 43 animals (43/370, Prevalence (*P*%) = 11.6%) were positive to *S. scabiei* antibodies with higher seroprevalence values in sows (35/151, *P* = 23.2%) if compared to fattening pigs (8/219, *P* = 3.6%). A higher seroprevalence was recorded in farms with poor or moderate scores (*P* = 100% and *P* = 64.3%, respectively) if compared to those with higher sanitary score (*P* = 44.4%). Statistical analysis revealed that production category and biosecurity score were significantly associated with the mite infestation. Sarcoptic mange can lead to significant economic losses in pig farming; besides, the zoonotic risk for human infestation due to handling of pig or carcasses should be considered.

## Introduction

Sarcoptic mange is a parasitic skin disease caused by the burrowing mite *Sarcoptes scabiei* (phylum Arthropoda, family Sarcoptidae), that lives in the epidermal layer of the host’s skin. This parasite is notable for its multiple host-adapted variants (“*varietates*”) within the same species. While *S. scabiei* has been reported in over 150 mammalian species, individual variants typically exhibit some degree of host preference, although cross-infection may occur (Arlian and Morgan 2017; Moroni et al. 2022). Animal-derived variants of *S. scabiei* could be responsible for human cases of zoonotic scabies, also known as “pseudoscabies”, a condition usually presenting as a self-limiting disease with a short incubation period and transient clinical signs (Moroni et al. 2022). Recently, sarcoptic mange was classified as a neglected tropical disease by the World Health Organization (WHO) (El-Moamly et al. 2021).

Transmission occurs mainly through prolonged direct contact and, in rare cases, indirectly through fomites, considering that *Sarcoptes* mites can survive in the environment for approximately 24–36 h under typical conditions, with survival extending up to about 5 days under optimal temperature and relative humidity (Arlian and Morgan 2017). Infestation by *S. scabiei* can cause dermatitis, with lesion distribution depending on the host species, and may also be associated with secondary bacterial skin infections. Clinical signs vary according to the age, immunocompetence and other physiological aspects of the host, and also the number of mites (Swe et al. 2014; Haas et al. 2015). In pigs, lesions typically appear in the ear area and spread progressively to other parts of the body developing into erythematous papules. Some animals may develop a chronic form of the disease, characterised by rough and thick skin, with crusts containing *Sarcoptes* mites. The associated itchiness usually develops between two and 11 weeks after infection and worsens due to a hypersensitivity response. In lactating sows, this can lead to marked restlessness and compromised maternal behaviour, resulting in insufficient nursing (Rambozzi et al. 2007; Goyena et al. 2013; Guillot et al. 2023). While sows primarily transmit the disease to their offspring, the role of boars as reservoirs should not be underestimated, particularly if they are not treated periodically (Genchi and Kramer 2000; Damriyasa et al. 2004).

Sarcoptic mange can lead to significant economic losses in pig farming (Bornstein and Wallgren 1997; Damriyasa et al. 2004). This is primarily due to reduced growth performance, lower feed conversion efficiency, impaired sow productivity, and increased piglet mortality from crushing. In the past, the main goal was to maintain an acceptable disease status to limit or eliminate the economic impact (Smets and Vercruysse 2002). However, in modern pig farming it is feasible to progress from disease control to disease eradication on a large scale (Heinonen et al. 2000). Effective disease control can indeed improve piglet growth performance, reduce average feed consumption in sows, shorten the interval between weaning and oestrus, and increase the annual number of weaned piglets per sow, ultimately leading to a recovery of treatment costs (Smets et al. 1999; Elbers et al. 2000; Mercier et al. 2002; Kessler et al. 2003; Laha 2015).

Sarcoptic mange has been reported in breeding sows and fattening pigs in every country where pig farming is practised. Worldwide, between 27.9 and 86.6% of pig herds were positive for *S. scabiei* with prevalence values from 8.3 up to 58% (Alonso de Vega et al. 1998). Prevalence estimates report that between 50 and 95% of herds in Europe are infested with *S. scabiei* mites (Cargill et al. 1997). Information on the prevalence of *S. scabiei* var. *suis* in domestic pigs in Italy is limited and inconsistent (Genchi and Kramer 2000; Galuppi et al. 2007; Maioli et al. 2015). However, recent serological surveys have demonstrated the widespread presence of this ectoparasite among free-ranging pig populations in France, Sweden, Italy, and Spain (Haas et al. 2015, 2018; Villa et al. 2023), suggesting that wild boars could also pose a risk to breeding pigs, since their presence in suburban and urban areas, coupled with the expansion of outdoor pig farming systems, increases the likelihood of *Sarcoptes* mites being transmitted to domestic pigs (Wu et al. 2012; Villa et al. 2023).

Due to the current lack of data on circulation of *S. scabiei* var. *suis* in pigs raised in Italy, this study aimed to assess its seroprevalence in selected fattening pig and sow farms located in the Lombardy region in northern Italy, where most of the total national pig herd is concentrated. The study highlights the usefulness of serological diagnostic methods for monitoring herd health status and for supporting the control of *S. scabiei* at the farm level.

## Materials and methods

### Sampling and data collection

Animal sampling was carried out as previously described for an epidemiological investigation on *Toxoplasma gondii* and *Neospora caninum* infection in pigs (Gazzonis et al. 2018; Villa et al. 2022) between November 2015 and October 2016 in Lombardy, one of the most suitable regions in northern Italy for intensive pig farming. A minimum sample size of 139 fattening pigs and 139 sows was determined by Epitools Epidemiological Calculators (www.epitools.ausvet.com.au), considering the population of fattening pigs and sows in the Lombardy region (1,076,334 and 225,088 individuals, respectively) (National Zootechnical Database, https://www.vetinfo.sanita.it/), a 10% expected prevalence, a 95% confidence level, and a 5% desired absolute precision. A total of 370 blood samples were collected at five slaughterhouses from 219 fattening pigs and 151 sows, originating from 15 to 11 conventional farms, respectively. On three farms (Farm 2, Farm 13, and Farm 17), it was possible to obtain samples from both the animal production categories. The average number of animals sampled per farm was 16 (range: 3–34). During the slaughter line, blood was collected from the jugular vein of each animal and transferred into sterile, anticoagulant-free tubes, which were then transported to the laboratory within a few hours after sampling. Upon arrival, samples were centrifuged (15 min, 2120x*g*) and sera were aliquoted into Eppendorf tubes and subsequently stored at − 20 °C until further serological analyses. At the time of sampling, data regarding farm management practices were recorded and a biosecurity score was assigned to each farm. Data were acquired by interviewing the farmer. The score was based on criteria related to the application of specific sanitary measures (i.e., application of a health management program, vaccination protocols, standard protocols for quarantine for imported animals, protocols for visitors/transporters, sanitary protocols for operators, application of an all-in/all-out system) and ranged from 1 (poor), 2 (moderate), and 3 (optimal) (Gazzonis et al. 2018).

### Serological analysis

Sera samples were analysed for the detection of anti-*S. scabiei* var. *suis* antibodies using a commercial indirect ELISA kit (*Sarcoptes*-ELISA 2001^®^ Pig, Afosa GmbH, Germany), following the manufacturer’s instructions. The kit specifications indicate a sensitivity of 94% and a specificity of 97% in domestic pigs. Specifically, the test uses *Sarcoptes* mites derived from pigs as antigen and is classified as the most sensitive among the indirect ELISA tests available on the market that are validated for pig samples (Löwenstein et al. 2004; Haas et al. 2015).

To calculate the percentage (P) of the optical density of the control sera, the following formula, provided by the kit manufacturer, was used: P = (OD_NC_ x 100)/OD_PC_. The results were considered correct with the following reference values: positive control serum 2.8 < OD_PC_ < 0.8; negative control serum *P* < 20; null value (sample dilution buffer) OD < 0.1. After obtaining the average of the optical density of the positive and negative controls (OD_PC_, OD_NC_), the average of the optical density of the negative controls was subtracted from the average of the optical density of the positive controls (OD_PC, corr_) and from the optical density of the samples (OD_Sample, corr_.). Subsequently, the test results (TE) were calculated according to the following formula: TE = (OD_Sample, corr_ x 100)/OD_PC, corr_. Samples with TE < 16 were considered negative, samples with TE between 16 and 24 were considered inconclusive, while samples with TE > 24 were considered positive.

### Statistical analysis

Seroprevalence of *S. scabiei* infection was calculated at the individual and farm levels according to the production category and sanitary score (Bush et al. 1997). A farm was considered positive if at least one sampled animal scored positive. Serological data were analysed to determine if production category and sanitary score could be predictors of *S. scabiei* infection; the farm was considered as the statistical unit. Overall, 26 observations were included in the analysis; indeed, the only three farms where both sows and fattening pigs were sampled, were defined as independent units, since management varies also within the same farm according to the production category. Generalized linear models (GLMs) with negative binomial distribution were performed to verify the influence of production category and sanitary score on *S. scabiei* infection. The intra-herd seroprevalence was considered as the dependent variable; the variables production category and sanitary score were entered as independent variables in the univariate model. All variables showing a *p value* < 0.1 were entered in multivariate models developed through a backward selection procedure (significance level to remove variables from the model *p value* = 0.05), based on Akaike’s information criterion (AIC) values. Statistical analysis was performed using IBM SPSS Statistics for Windows version 25.0 software (IBM Corp., Armonk, NY, USA).

## Results

At the individual level, 43 out of 370 animals tested positive for *S. scabiei* antibodies (*P* = 11.6%, 95% CI: 8.5–15.3). A higher seroprevalence was observed in sows (35/151, *P* = 23.2%, 95% CI: 16.7–30.7) than in fattening pigs (8/219, *P* = 3.6%, 95% CI: 1.6–7.1). Intra-herd seroprevalence of the mite infestation ranged from 2.5% to 100%, particularly from 3.3% to 100% in sows whereas in fattening pigs it shows lower variation (from 4% to 20%) (Table 1).

**Table 1:** Prevalence of antibodies anti-*Sarcoptes scabiei* var. *suis* in fattening pigs and sows on each farm examined in northern Italy

ID Farm	Fattening pigs		Sows		Total	
	*N*° positive/examined animals	*P*% (95% CI)	*N*° positive/examined animals	*P*% (95% CI)	*N*° positive/examined animals	*P*% (95% CI)
1	–	–	6/7	85.7 (42.1–99.6)	6/7	85.7 (42.1–99.6)
2	0/30	0 (0.0–11.3.0.3)	2/4	50 (6.7–93.2)	2/34	5.8 (0.7–19.7)
3	0/12	0 (0.0–24.2.0.2)	–	–	0/12	0 (0.0–17.6.0.6)
4	0/10	0 (0.0–27.7.0.7)	–	–	0/10	0 (0.0–27.7.0.7)
5	0/18	0 (0.0–17.6.0.6)	–	–	0/18	0 (0.0–17.6.0.6)
6	0/18	0 (0.0–17.6.0.6)	–	–	0/18	0 (0.0–17.6.0.6)
7	–	–	2/30	6.7 (0.8–22.1)	2/30	6.7 (0.8–22.1)
8	–	–	5/5	100 (47.8–100)	5/5	100 (47.8–100)
9	1/5	20 (0.5–71.6)	–	–	1/5	20 (0.5–71.6)
10	1/10	10 (0.2–44.5)	–	–	1/10	10 (0.2–44.5)
11	1/15	6.7 (0.2–31.9)	–	–	1/15	6.7 (0.2–31.9)
12	–	–	4/4	100 (40–100)	4/4	100 (40–100)
13	0/10	0 (0.0–27.7.0.7)	1/30	3.3 (0.08–17.2)	1/40	2.5 (0.06–13.2)
14	–	–	4/4	100 (40–100)	4/4	100 (40–100)
15	–	–	5/30	16.7 (5.6–34.7)	5/30	16.7 (5.6–34.7)
16	–	–	0/30	0 (0.0–11.3.0.3)	0/30	0 (0.0–11.3.0.3)
17	1/25	4 (1–20.3)	4/4	100 (40–100)	5/29	17.2 (5.8–35.8)
18	2/10	20 (2.5–55.6)	–	–	2/10	20 (2.5–55.6)
19	0/18	0 (0.0–17.6.0.6)	–	–	0/18	0 (0.0–17.6.0.6)
20	0/10	0 (0.0–27.7.0.7)	–	–	0/10	0 (0.0–27.7.0.7)
21	2/18	11.1 (3.1–32.8)	–	–	2/18	11.1 (3.1–32.8)
22	0/10	0 (0.0–27.7.0.7)	–	–	0/10	0 (0.0–27.7.0.7)
23	–	–	2/3	66.7 (9.4–99.2)	2/3	66.7 (9.4–99.2)
**Total**	8/219	3.6 (1.6–7.1)	35/151	23.2 (16.7–30.7)	43/370	11.6 (8.5–15.3)

*P*%, prevalence, CI, confidence interval

At the farm level, seropositivity was detected in 15 out of 23 farms (*P* = 65.2%, 95% CI: 47.1–86.8). This was specifically found in 10 out of 11 farms (*P* = 90.9%, 95% CI: 58.7–99.8) housing sows and in six out of 15 farms (*P* = 40%, 95% CI: 16.3–67.7) housing fattening pigs. Considering the sanitary score, a higher seroprevalence was recorded in farms with poor or moderate scores (*P* = 100% and *P* = 64.3%, respectively) if compared to those with higher sanitary score (*P* = 44.4%).

Univariate analysis revealed that both the variables considered (production category and biosecurity score), were significantly associated with *S. scabiei* infection, and were therefore included in the final multivariate model. Multivariate analysis showed that both variables were significantly associated with the mite infestation. Indeed, farms raising sows had a higher risk of seropositivity to *S. scabiei* (OR = 1.6, *p* = 0.000) than those raising fattening pigs. Farms with a high (OR = 0.7, *p* = 0.038) or moderate (OR = 0.7, *p* = 0.050) biosecurity score showed a lower risk of mite infestation than those with a low score (Table 2).

**Table 2:** Multivariate analysis of the risk factors for *Sarcoptes scabiei* var. *suis* infestation as determined by antibodies detection on farms in northern Italy housing fattening pigs and sows

Response variable	Category	*N*° positive/examined farms	*P*% (95% CI)	Odd Ratio (95% CI)	*p* value	Akaike information criterion
Production category	Sows	10/11	90.9% (58.7–99.8)	1.6 (1.3–1.9)	**0.000**	23.38
	Fattening pigs	6/15	40.0% (16.3–67.7)	1		
Sanitary score	1	3/3	100 (29.2–100)	1		
	2	9/14	64.3 (35.1–87.2)	0.7 (0.5–0.9)	**0.038**	
	3	4/9	44.4 (13.7–78.8)	0.7 (0.5–1.0.5.0)	**0.050**	

*P*%, prevalence, CI, confidence interval

## Discussion

This study provides update data on the prevalence of *S. scabiei* var. *suis* on intensive pig farms in northern Italy, improving the knowledge of the seroprevalence and risk factors of this ectoparasite of relevance for swine farming.

At the individual level, a seroprevalence of 11.6% was observed. Only few serological studies have assessed the circulation of this mite in pigs. In Italy, a study conducted in fattening pigs of 9–10 months of age from farms in central and northern regions (Emilia Romagna, Lombardia, Piemonte and Umbria) reported a seroprevalence 15.5% and 22.8% measured by ELISA tests on sera and meat juice, respectively (Galuppi et al. 2007). In Europe, a survey conducted in Austria reported a seroprevalence measured by ELISA of 28.2%, highlighting the potential for subclinical presence of *Sarcoptes* mites in closed herds (Löwenstein et al. 2006b). By contrast, research based on direct detection methods, such as skin scrapings, has been carried out more frequently worldwide, showing variable prevalence values ranging from 18.3 up to 58%. In Italy, individual prevalence values of 5.7% (Galuppi et al. 2007) and 13.7% (Maioli et al. 2015) up to 44% to 62% (Genchi and Kramer 2000) were reported by ear or skin scraping. In Spain, two studies reported prevalence values of 33.5% (75/224) and 25.6% (337/1318), respectively, based on the direct microscopic examination of ear scrapings (Gutiérrez et al. 1996; Alonso de Vega et al. 1998). These discrepancies may be attributed to the production category sampled, the laboratory procedures employed for the microscopic detection of the mites, and the geographic area examined (Alonso de Vega et al. 1998; Damriyasa et al. 2004; Kabululu et al. 2015).

The seroprevalence of 65.2% at farm level from this study revealed that *S. scabiei* is widespread among pig herds. A recent report on Italian pigs showed a lower herd prevalence value (*P* = 19.6%) by analysing pools of ear scrapings (Maioli et al. 2015), but previous studies reported prevalence values at the farm level from 50% (Galuppi et al. 2007) up to 86–100% (Genchi and Kramer 2000) by serology and ear scraping, respectively. Among the reasons for the difference in the observed prevalence values, the main is the different analytical methods used: although ear or skin scraping offers high specificity by the direct observation of live mites, its sensitivity is lower than serology (Debnath et al. 2020), particularly in subclinical and chronic cases. Besides, direct microscopic examination cannot distinguish between active and inactive infestations, since dead mites can persist for up to 15 days after acaricidal treatment (Maioli et al. 2015). In any case, Galuppi et al. (2007) compared four diagnostic tests (average dermatitis score, skin scraping, ELISA on serum and on meat juice) for the detection of *S. scabiei* in swine revealing poor or no correlations and suggesting their application in association for control programmes.

Considering the production category, sows were infested more frequently than fattening pigs (*P* = 23.2% and *P* = 3.6%) and farms housing sows showed a significantly higher risk of *S. scabiei* infestation than farms housing fattening pigs (*P* = 90.9% and *P* = 40.0%). In this regard, the longer lifespan of sows may increase their exposure to the parasite throughout the production cycle; besides, physiological stress associated with farrowing and lactation can also cause temporary immunosuppression making sows more susceptible to parasitic diseases. Among managerial factors, housing dry and nursing sows in the same units poses an additional risk, since the absence of physical separation facilitates contact between them and potential parasite transmission; moreover, the straw bedding, often used in farrowing pens, provides a suitable environment for the parasite to survive by creating a favourable microclimate that allows mites to persist longer off the host, thereby increasing the likelihood of transmission between sows and from sows to piglets (Damriyasa et al. 2004; Guillot et al. 2023).

On the other side, the low prevalence observed in fattening pigs may reflect high biosecurity standards typically adopted in this category, alongside the regular application of treatment protocols commonly applied during the growing phase, which could reduce the early-life infections acquired from the mother at slaughter age. In this context, most fattening farms in northern Italy are part of Protected Designation of Origin (PDO) and Protected Geographical Indication (PGI) circuits, which require strict hygiene, health, disease prevention and control measures to ensure optimal slaughter weight and minimise economic losses (Bottacini et al. 2018; Allievi et al. 2024).

However, as detailed information on farm-level treatment protocols was not available, the relative contribution of management practices versus biosecurity conditions could not be fully assessed. Besides, it should also be considered that the sanitary score used in this study does not specifically account for factors directly associated with *S. scabiei* transmission or treatment efficacy, which should be considered when interpreting these results.

Regarding herd management practices, seroprevalence declined significantly as biosecurity improved, from 100% in farms with a poor score to 64.3% and 44.4% in those with moderate and optimal scores, respectively. These results imply that inadequate sanitary and hygiene measures facilitate the transmission of *S. scabiei*, particularly in densely populated environments with restricted outdoor access. On the contrary, farms with well-structured management systems can maintain high biosecurity levels and promote animal welfare reducing the risk of parasite circulation (Alarcón et al. 2021; Pettersson et al. 2021).

Since sarcoptic mange is recognised as an emerging zoonotic disease in wild animal species (Valldeperes et al. 2021), it should also be considered the potential risk at the interface between wildlife and livestock, to mitigate the potential for cross-species transmission (Pence and Ueckermann 2002; Haas et al. 2018; Sanno et al. 2021). Indeed, although sarcoptic mange is primarily transmitted through direct contact, it can also be transmitted indirectly between different host species; spillover events are mostly reported among closely related species or in predator-prey systems, whereas transmission between more distantly related hosts usually results in self-limiting infestations (Oleaga et al. 2008; Iacopelli et al. 2020; Valldeperes et al. 2021). In this context, as previously evidenced, wild boars, but also the red fox and the Iberian wolf, may play a key epidemiological role in linking wildlife and domestic cycles (Moroni et al. 2021). Since according to recent surveys infestation by *Sarcoptes* is widespread in wild boars, also in animals captured in areas with numerous pig farms (Haas et al. 2018; Villa et al. 2023), infested wild boars may pose a risk for domestic pigs, particularly in areas where their habitat overlap or in herds with inadequate biosecurity measures (Oleaga et al. 2008; Moroni et al. 2021).

Furthermore, even though *S. scabiei* var. *suis* is generally considered host-specific, zoonotic transmission to humans may still occur through close contact with infested pigs or carcasses, representing an occupational risk for farmers, veterinarians, and abattoir workers (Moroni et al. 2022).

The economic impact of sarcoptic mange in pig farming is considerable, since infestations are associated with reduced weight gain, impaired feed conversion, lower reproductive performance, and increased veterinary costs (Arends et al. 1990). Therefore, routine monitoring and early detection are crucial for the timely implementation of acaricide treatments. Due to the subclinical or chronic nature of sarcoptic mange in pigs, traditional diagnostic methods, i.e. clinical observation of animals or direct mite detection via skin scraping, may significantly underestimate the true prevalence of infestation. In contrast, serological tests as the ELISA offer significant advantages for herd-level surveillance: indeed, this approach is applicable on a large scale, time-efficient, and capable of detecting asymptomatic cases that might otherwise go unnoticed (Bornstein and Wallgren 1997; Kessler et al. 2003). However, it should be noted that, despite their advantages, serological methods may occasionally have lower sensitivity than direct detection techniques, e.g., in acute cases, due to the time required for seroconversion, with antibodies appearing weeks after infestation and therefore early false‑negative results (Löwenstein et al. 2006a). In some cases, doubtful results should be confirmed through clinical examination and parasitological methods.

One of the main challenges in controlling *S. scabiei* var. *suis* is the transmission from infested sows to suckling piglets. Treating pregnant sows just before they are moved to the farrowing unit can therefore reduce infestation in both the sows and their piglets. In addition, regular serological screening with ELISA is useful for surveillance and for documenting infestation-free status; however, maintaining such a status depends on the implementation of effective biosecurity measures that prevent the introduction of *S. scabiei*, as serological screening alone is not sufficient to ensure disease freedom (Laha 2015). However, it is still essential to monitor the spread of sarcoptic mange during the post-weaning and fattening periods, since mites can reduce growth rates and welfare, induce pruritus resulting in increased scratching time, and cause papular dermatitis, resulting in significant economic losses at the farm level (Davis and Moon 1990; Davies 1995).

Despite the regular use of drugs for the control of parasitic diseases in modern pig farming, these results demonstrated that cases of sarcoptic mange can still occur. This is probably due to the use of non-standardised protocols, the administration of incorrect dosages of the compounds, the introduction of new animals to the farm without observing an isolation period or treating them with the appropriate molecules, and potential environmental contamination. In fact, to optimally control the mite, it is necessary to consider that, since antiparasitic drugs are generally ineffective against eggs, treatment should be repeated 14 days after the initial treatment to coincide with the parasite’s life cycle. Moreover, it should be considered that mites can survive outside the pig for up to 12 days in temperatures ranging from 7 °C to 18 °C and relative humidity levels between 65% and 75% (Jacobson et al. 1999, 2000; Laha 2015). In this context, increasing concerns regarding the environmental impact of these compounds and the potential risk of reduced efficacy with repeated use highlight the importance of sustainable control strategies, based on integrated approaches combining surveillance, targeted treatment, and strict biosecurity measures.

Among the limitations of this study, only a limited number of randomly selected farms from the intensive pig farming system of the Lombardy region in northern Italy were included; the sampling was performed approximately ten years ago, and even if no major changes in the pig production system have occurred during this period, recent developments in herd health management may still have influenced the current epidemiological situation. The study is based exclusively on serological data, which reflects exposure to *S. scabiei* rather than confirming active infestation. Since no direct clinical examination or parasitological confirmation at the animal level was performed, it is not possible to distinguish between current and past infections. Furthermore, detailed farm-level information, was not available, limiting a deeper interpretation of the observed prevalence values. Therefore, the results should be interpreted primarily as baseline epidemiological data on exposure to *S. scabiei* in Italian pig populations at the time of sampling. For a more comprehensive and up-to-date assessment of the current situation and associated risk factors, further studies incorporating longitudinal sampling across a larger number of farms, integration of clinical and serological data, and detailed farm management data are needed.

Integrating regular serological screening into routine herd health monitoring programmes provides a cost-effective and sensitive approach for managing *S. scabiei* infestations. Serological testing can be a useful tool for diagnostics and herd-level surveillance, enabling the identification of infected herds and supporting the timely implementation of targeted control measures. When combined with appropriate biosecurity measures, strategic use of acaricides, and continued training for farmers and veterinarians, this approach can indirectly contribute to improved sustainable control, reduced transmission, mitigation of the economic burden associated with sarcoptic mange in pig farming, and a reduction in potential zoonotic risk through enhanced management and biosecurity practices.

## Conclusions

This study demonstrated the circulation of *S. scabiei* var. *suis* in intensive pig farming systems in northern Italy. The moderate individual prevalence and the high farm-level prevalence indicated the need to adopt targeted control measures using appropriate pharmacological treatments and strict sanitation protocols, while also considering the sustainability of pig farming systems. In this context, serological surveys are essential to obtain a comprehensive epidemiological overview of sarcoptic mange in pigs, focusing on its prevalence and the associated economic impact on the pig industry. Finally, due to the zoonotic potential of this ectoparasite, adequate risk communication strategies are required to raise awareness among personnel involved in the food production chain.

## Data Availability

The data that support the findings of this study are available from the corresponding author upon reasonable request.
